# Exome sequencing detected an extremely rare case of foetal onset familial haemophagocytic lymphohistiocytosis type 5 presenting with hydrops foetalis

**DOI:** 10.1186/s12920-021-00897-z

**Published:** 2021-02-16

**Authors:** V. Thadchanamoorthy, M. T. R. Jayatunga, Kavinda Dayasiri, E. Jasinge, M. L. M. Jinnah, C. Pereira, V. Skrahina, Markandu Thirukumar

**Affiliations:** 1 Department of Clinical Sciences, Faculty of Health Care Sciences, Eastern University of Sri Lanka, Chenkaladi, Sri Lanka; 2 Department of Neonatology, Teaching Hospital, Batticaloa, Sri Lanka; 3 Department of Paediatrics, Base Hospital, Mahaoya, Sri Lanka; 4 Department of Biochemistry, Lady Ridgeway Hospital, Colombo, Sri Lanka; 5 CENTOGENE AG, Rostock, Germany

**Keywords:** Familial, Hemophagocytic lymphohistiocytosis, *STXBP2* gene, Jaundice, Genetic counselling

## Abstract

**Background:**

Familial hemophagocytic lymphohistiocytosis (FHL) is a genetically heterogeneous autosomal recessive hyper-inflammatory syndrome which needs early accurate diagnosis and appropriate treatment to prevent complications and early mortality. Recently, it was reported that mutations in *STXBP2* gene are linked to FHL type 5 (FHL-5).

**Case Presentation:**

We report a Sri Lankan neonate who presented with low Apgar scores at birth, abdominal distension, and hepatosplenomegaly, followed by lethargy, poor sucking and rapid decompensation with wide spread activation of inflammation within 48 h of birth. Her elder sibling also had a similar presentation during early neonatal period and deceased at two weeks of age with no diagnosis. Unfortunately, the index case deceased at 14 days of age following multi-organ dysfunction and severe metabolic acidosis. Targeted gene panel followed by reflex exome sequencing revealed a novel likely pathogenic homozygous variant in the *STXBP2* gene (NM_001272034.1:c.1141-2A > G) which confirmed the diagnosis of autosomal recessive FHL-5.

**Conclusion:**

Early diagnosis of FHL type 5 using genetic analysis and timely treatment are difficult in the absence of family history due to a wide spectrum of clinical manifestations. However both early diagnosis and treatment doesn’t alter the long term prognosis. So genetic counselling would be the better option.

## Background

Familial hemophagocytic lymphohistiocytosis (FHL) is a rare and potentially fatal autosomal recessive hyper-inflammatory syndrome [1]. FHL is caused by genetic mutations resulting in defective cell cytotoxicity. There are five disease related genes: mutation in chromosome 9q (FHL1/ type 1), *PFR1* (FHL2/ type 2), *UNC13D* (FHL3/ type3), *STX11* (FHL4/type 4) and *STXBP2* (FHL5. type 5) [2]. But four genes in which pathogenic variants are causative have been identified: FHL types 2, 3 and 4 are associated with defective function of perforin, Munc13-4 and syntaxin-11 respectively, whilst type 5 (*STXBP2)* is associated with a defect in Munc18-2 [3, 4]. The defective genes in FHL lead to ineffective T lymphocyte and natural killer cell degranulation by inhibition of cytotoxic granule components impairing their fusion machinery [5]. Ultimately, the disease is characterized by inefficient removal of target cells and uncontrolled activation of macrophages and T cells [1]. The incidence of FHL is about 0.12 to 0.15 per 100,000 children per year [1, 5, 6]. FHL-5 secondary to causative variants of *STXBP2* accounts for 10% of all FHL cases [1, 5, 6]. FHL type 5 due to mutation by causative variants of STXBP2 with neonatal presentation is extremely rare [8] and most children present between 2 and 6 months of age [9]. Due to atypical and wide range of presentation of FHL, most neonates who do not have a positive family history are diagnosed either late in the course of the disease or following death [10]. FHL is clinically characterized by recurrent fever, hepatosplenomegaly, cytopenias, hypertriglyceridemia, hypofibrinogenemia, hyperferritinemia and hemophagocytosis in bone marrow and the clinical features mimic severe sepsis [11]. In the newborn with hydrops foetalis, the disease resembles congenital infections. The mortality of untreated patients can be as high as 95%. The recognized treatment modalities include immune-chemotherapy for disease control and hematopoietic stem cell transplantation. The authors report a neonate with foetal onset of FHL5 disease with hydrops foetalis and a novel homozygous likely pathogenic variant in the *STXBP2* gene.

## Case presentation

**Fig. 1:**
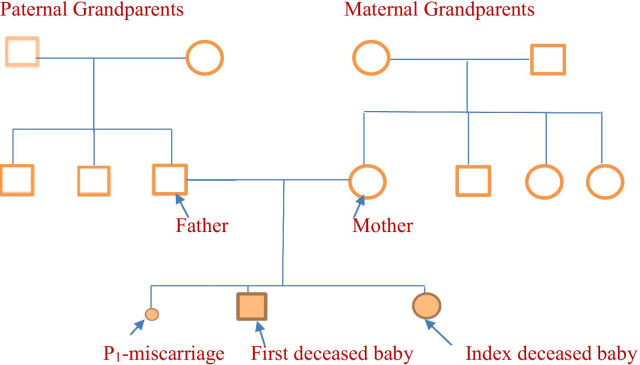
Pedigree from the FHL families with STXBP2 gene mutation

The index case is a female newborn found to have low Apgar scores and abdominal distention at birth. She was the product of third pregnancy of non-consanguineous parents. The first pregnancy ended as a spontaneous-onset first trimester miscarriage. Her elder child, born following second pregnancy, died during the neonatal period with no diagnosis. The elder sibling had a similar presentation during the early neonatal period and later developed severe sepsis and metabolic acidosis (Fig. 1).

The index child was born by elective caesarian section weighing 3 kg at term. Mother did not report of any medical disorders or infections complications during the pregnancy. After low Apgar scores improved following initial resuscitation, she continued to have mild tachypnea and abdominal distension. She had poor sucking, fever, neonatal jaundice and marked hepatosplenomegaly on day 1.

The baby was treated as for severe sepsis with intravenous antibiotics despite negative septic screen. However, given the previous unexplained neonatal death, this child was thoroughly investigated for a differential diagnosis including metabolic disorders. Over the course, the baby deteriorated with worsening of jaundice and needed ventilatory care.

Her liver functions were abnormal with evidence of widespread activation of inflammation: INR (International normalized ratio)—6.23, ALT-586 u/L, AST-5865 u/L, serum bilirubin 362 micromol/L, GGT-248u/L, serum albumin-2.2 g/dl, APTT – 48 s. Renal functions were also deranged: blood urea—146 mg/dL, serum creatinine—2.2 mg/dL). Haematological parameters showed progressive increase in white blood cells (17 × 10^3^ mm/L with neutrophils -30%), thrombocytopenia (platelets—8 × 10^3^ mm/L), and anemia (haemoglobin—6.3 g/dL). C-reactive protein (CRP) was 116 mg/dL. Blood smear showed features of infection with toxic granules and pancytopenia. Metabolic investigations showed: lactic acidosis and increased urinary excretion lactic acid and 4-hydroxyphenyllactic acid. Serum alpha-fetoprotein was very high (14900 ng/ml). TORCH screening was negative. Screening for Hepatitis B and hepatitis C was negative. Ferritin and triglyceride were not performed. Blood and CSF cultures remained negative while urine culture grew *Klebsiella*. Biochemical studies of cerebrospinal fluids were normal. Ultrasound chest and abdomen showed pleural effusion and ascites respectively and the findings were in keeping with hydrops fetalis. Brain ultrasound was normal.

Despite intensive management with ventilatory care, inotropes, blood and its derivatives, and potent intravenous antibiotics, the patient deteriorated leading to multi-organ dysfunction and severe metabolic acidosis and deceased on day 14 while receiving ventilator care.

Given the clinical picture of the patient, genetic analysis of an extended genetic panel was requested at CENTOGENE AG. Genomic DNA was enzymatically fragmented and regions of interest were enriched using DNA capture probes targeted against the coding regions along with flanking ± 10 intronic bases and known pathogenic/likely pathogenic variants of the panel genes. The libraries were subsequently sequenced on an Illumina platform.

Initial evaluation of a gene panel was negative, thus, the analysis was extended to other genes from the exome. A homozygous splicing variant in the *STXBP2* [Chr19(GRCh37)] gene NM_001272034.1:c.1141-2A > G was detected. The variant is extremely rare and absent in control patients. In silico tools predict that this variant disrupts the highly conserved acceptor splice site of exon 14. This variant is classified as likely pathogenic based on the American College of Medical Genetics and Genomic guidelines (ACMG). The following criteria for variant classification were applied: PVS1 (null variant) and PM2 (absent or very rare in controls, i.e. gnomAD) [12]. The annotation of the variant and the predictions tools in silico used are present in Table 1. For splicing mutations, Ada- and RF-scores, together with conservation were considered. At the time of the initial report, we were also using SSF, MaxEnt, HSF prediction tools that all supported the likely splice effect as reported (threshold differences are > 5% for SSF, > 15% for MaxEnt or > 10% for HSF) (Table 2).

**Table 1:** The annotation of the reported variant and the predictions tools in silico

Variant coordinates	In silico prediction tools	Deleterious threshold
Chr19(GRCh37):g.7709498A > G	Ada Score: 1.0000	Ada score < 0.6
NM_001272034.1:c.1141-2A > G	Rf score: 0.9360	RF score < 0.6
	phyloP100way: 7.39	< 1.5

**Table 2:** Clinical and genetic information of deceased babies

Manifestations	First baby ( male)	Second baby (female)
Birth weight	3.2 kg	3 kg
Apgar at 5minutes	10	08
Abdominal distension at birth	Slight distention	Significant distension
Tachypnea, poor sucking, less activity	Developed on day 03	Since birth
Jaundice	Developed on day 03	Day 01
Hepatomegaly	4 cm on day 03	8 cm at birth
Splenomegaly	2 cm on day 03	3 cm at birth
Multi-organ dysfunction	Developed on day 10	Day 05
Septic screening (FBC,CRP)	Altered at day 03	Altered on day 02
Liver Function Tests		
AST	–	5865 u/L
ALT	–	586 u/L
Serum bilirubin	–	362 micromol/L
Serum albumin	–	2.2 g/dl
GGT	–	248u/L
INR	–	6.23
APTT	–	48 s
Renal Function Tests		
Blood urea	–	146 mg/dL
Serum Creatinine	–	2.2 mg/dL
Urine culture	Klebsiella	Klebsiella
Blood &CSF cultures	Negative	Negative
TORCH screening	Negative	Negative
Screening Hepatitis B& C	Negative	Negative
Ultrasound abdomen and chest	Hepatosplenomegaly with mild ascites on day 03, but no pleural effusion	Hepatosplenomegaly with gross ascites and pleural effusion on day 01
Liver biopsy	Acute hepatitis with liver cell necrosis	Not done
Deceased	On day 14	On day 14
Genetic study	Not performed	Homozygous, *STXBP2* gene NM_001272034.1:c.1141-2A > G

Parents were offered genetic studies to confirm heterozygosity of the variant and arrangements were made to follow up with genetic counseling and prenatal diagnosis.

Both babies presented with similar clinical features in the early neonatal period, but index baby exhibited clinical features at birth that the first deceased baby discharged on day 2 following neonatal assessment with normal ultrasound abdomen, only showed after readmission on the following day due to poor sucking and mild jaundice. Table 1 described the clinical and genetic information of both deceased babies.

## Discussion and conclusions

Familial hemophagocytic lymphohistiocytosis is characterized by uncontrolled inflammation. Fetal onset FHL is considered to be the most severe form and carries high mortality [10]; however due to extreme rarity, disease characteristics of fetal onset neonatal FHL is not well described [13].

FHL is caused by genetic mutations resulting in defective cell cytotoxicity and it is divided in five different subtypes, depending on the affected gene [2]. Our newborn had a novel homozygous likely pathogenic variant in the *STXBP2* gene (type 5) which is associated with defect in Munc18-2 [3, 4]. *STXBP2* is a part of the Sec/ Munc proteins that are important for the assembly and disassembly of the SNARE (soluble N-ethylmaleimide-sensitive factor attachment protein receptor) complex and the control of membrane fusion. The exact role of *STXBP2* in the cytotoxic granule pathway is not presently described in depth, however, the previous studies detailed that the protein is also involved in the regulation of intracellular granule trafficking in the epithelial cells, neutrophils, and mast cells [14]. Deficiency of this protein leads to defective cytotoxic activity and regulation of epithelial cells and neutrophils, and removal of target cell and uncontrolled activation of macrophages and T lymphocytes which causes immune dysfunction [1].The uncontrolled inflammation in FHL is associated with very high interleukin 6, interleukin 8 and tumor necrosis factors 8 levels with markedly reduced natural killer cell function in the neonate [15].

Although clinical features in FHL usually present during the first year of life, the affected individuals might not reveal symptoms until later in childhood or even into adulthood [16]. Well-recognised clinical features of FHL include prolong fever, enlarged liver and/or spleen, skin rash, lymph node enlargement, breathing problems, kidney abnormalities, heart problems, and cytopenias. FHL is also associated with an increased risk for certain haematological malignancies such as leukemia and lymphoma [17, 18].

FHL5 typically presents within the first months or years of life and occasionally, in utero and is associated with atypical features such as severe diarrhoea, abnormal bleeding, and early onset neurological abnormalities including hearing loss [14, 19]. The index case exhibited symptoms at birth which include, poor sucking, less active, tachypneoa, hepatosplenomegaly, hydrops foetalis, cytopenia and jaundice. Subsequently prolong fever, septicaemia and bleeding tendency secondary to liver dysfunction and thrombocytopenia were observed. Although she did not have renal and cardiac abnormalities at birth, she developed metabolic acidosis and multi-organ failure with the progression of the disease.

The diagnosis of hemophagocytic lymphohistiocytosis (HL) can be established If 1 and/or 2 of below criteria are fulfilled: (1) a genetic test identifying a mutation in one of the genes known to be involved in FHL, (2) at least five out of the following eight clinical and laboratory features: fever, splenomegaly, pancytopaenia, hypertriglyceridemia, hypofibrinogenaemia, hyperferritinaemia, haemophagocytosis on bone marrow smear, spleen or lymph node biopsy, decreased or absent natural killer cell activity, and elevated blood levels of CD25 [19, 20]. The genetic study confirmed the diagnosis in our newborn.

Treatment depends on a number of factors, including the severity of symptoms, the age of onset, and the underlying cause of haemophagocytosis. Allogeneic hematopoietic cell transplantation is thought to be curative for FHL. Better outcomes have been observed following early marrow transplantation in confirmed or suspected patients with FHL [21]. Before the era of allogeneic hematopoietic cell transplantation, patients were usually treated with chemotherapy and/or immunotherapy such as corticosteroids, cyclosporine, etoposide, or anti-thymocyte globulin to destroy excess immune cells, and to counteract life-threatening hyper-inflammatory syndrome. Since the authors did not suspect the possibility of FHL at birth and the diagnosis was only made retrospectively based on genetic studies, these treatment options could not be offered to the reported patient.

All forms of FHL, whether treated early or not, carry a high mortality rate. The long-term prognosis of familial forms without treatment is poor, with a median survival of less than 2 to 6 months after appearance of symptoms. Even with proper treatment, estimated five-year survival would only be 21–26% in patients with FHL [22].

We offered genetic counselling with the help of multidisciplinary team which includes Consultant Genetics, Neonatologist, Obstetrician and Gynecologist, Psychiatrist and Chemical pathologist on regular time basis. They accepted the loss and now have a normal social life. Furthermore, we explained the various options to have a baby including adopting a baby. Although we offered the genetic studies on parents and explained the future intrauterine diagnosis, parents would like to wait 2 years to plan next pregnancy and are planning genetic studies, but their economic condition and religious background are barriers that prevent them to conduct further genetic studies at the moment. Till then, parents agreed to follow-up counselling with our team.

Early diagnosis of FHL type 5 is often difficult in the absence of family history and also due to a wide range of clinical presentations. Early diagnosis is crucial in timely treatment. Exome sequencing has the potential to genetically diagnose these patients with heterogeneous clinical presentations.

## Data Availability

The data that support the findings of this case report are available from Medical Records Department, Batticaloa Teaching Hospital, but restrictions apply to the availability of these data, which were used under license for the current report and so are not publicly available. Data are, however, available from the authors upon reasonable request and with permission of Medical Records Department, Batticaloa Teaching Hospital, Sri Lanka.

## Abbreviations

FHL: Familial Hemophagocytosis Lymphohistiocytosis
HL: Hemophagocytosis Lymphohistiocytosis
CSF: Cerebrospinal fluid
B/C: Blood culture
ALT: Alanine transaminase
AST: Aspartate transaminase
GGT: Gamma-glutamyl transferase
APTT: Activated Partial Thromboplastin Time
TORCH screen: Toxoplasmosis, other, rubella cytomegalovirus, herpes simplex, and HIV screen
